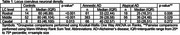# Younger age of onset and atypical clinical syndrome are associated with greater locus coeruleus degeneration in Alzheimer's disease

**DOI:** 10.1002/alz70855_103303

**Published:** 2025-12-24

**Authors:** Sara R Dunlop, Billie Matchett, Alexander Mannsbart, Fadi S Hanna Al‐Shaikh, Darren M. Rothberg, Josalen Ventura, Idaly Velez‐Uribe, Ranjan Duara, Neill R. Graff‐Radford, Dennis W. Dickson, Lea T. Grinberg, Melissa E. Murray

**Affiliations:** ^1^ Department of Neuroscience, Mayo Clinic Florida, Jacksonville, FL, USA; ^2^ Wien Center for Alzheimer's Disease and Memory Disorders, Miami Beach, FL, USA; ^3^ Department of Neurology, Mayo Clinic, Jacksonville, FL, USA; ^4^ Department of Laboratory and Medicine, Mayo Clinic, Jacksonville, FL, USA; ^5^ Memory and Aging Center, UCSF Weill Institute for Neurosciences, University of California, San Francisco, San Francisco, CA, USA

## Abstract

**Background:**

The locus coeruleus (LC) degenerates early in Alzheimer's disease (AD), with profound volume and neuronal loss. The loss of noradrenergic LC projection neurons results in alterations in attention, memory, and arousal, and constitutes the first site of neurofibrillary tangle accumulation preceding the onset of cognitive symptoms. We sought to investigate the relationship between LC neuronal density and clinical heterogeneity in a neuropathologically‐diagnosed series of AD cases.

**Methods:**

The FLorida Autopsied Multi‐Ethnic (FLAME) cohort database was queried for neuropathologically‐diagnosed AD cases with a clinical diagnosis of AD dementia or an atypical, non‐amnestic clinical syndrome (e.g., corticobasal syndrome, posterior cortical atrophy). AD cases were selected based on high AD neuropathologic change (ADNC) and lack of frontotemporal lobar degeneration or hippocampal sclerosis, resulting in 591 AD cases and an additional 74 none‐low ADNC controls. Databased clinical diagnosis for AD cases was classified as amnestic or non‐amnestic. Hematoxylin & eosin‐stained slides were digitized and neuronal density was quantified using an in‐house developed macro on 900µmx900µm region of interest using Aperio technology (Leica Biosystems). LC level was classified using neuroanatomic landmarks as rostral, middle, or caudal.

**Results:**

Control cases had greater neuronal density in the LC compared to AD cases at all three levels, *p* <0.05. In AD cases, we observed an association between age and neuronal density. Examining these differences along the rostrocaudal‐axis, we found that a younger age at symptomatic onset was associated with lower LC neuronal density in the rostral (correlation coefficient: 0.48; *p* <0.001) and middle LC (correlation coefficient: 0.28; *p* <0.001), no association between age at onset and neuronal density was observed at the caudal level. Stratifying our analysis by clinical syndrome, we found fewer neurons in the rostral and middle LC in non‐amnestic AD compared to amnestic AD (*p* = 0.006 and 0.029 respectively, Table 1). No significant differences between amnestic and non‐amnestic cases were observed in the caudal LC.

**Conclusions:**

The locus coeruleus, especially the rostral and middle levels, are especially vulnerable in young‐onset and atypical forms of AD. The loss of noradrenergic neuromodulation may contribute to the atypical symptomatology.